# Oral tolerance inhibits pulmonary eosinophilia in a cockroach allergen induced model of asthma: a randomized laboratory study

**DOI:** 10.1186/1465-9921-11-160

**Published:** 2010-11-23

**Authors:** Louis J Vaickus, Jacqueline Bouchard, Jiyoun Kim, Sudha Natarajan, Daniel G Remick

**Affiliations:** 1 Boston University School of Medicine, Department of Pathology and Laboratory Medicine, Boston, MA USA

## Abstract

**Background:**

Antigen desensitization through oral tolerance is becoming an increasingly attractive treatment option for allergic diseases. However, the mechanism(s) by which tolerization is achieved remain poorly defined. In this study we endeavored to induce oral tolerance to cockroach allergen (CRA: a complex mixture of insect components) in order to ameliorate asthma-like, allergic pulmonary inflammation.

**Methods:**

We compared the pulmonary inflammation of mice which had received four CRA feedings prior to intratracheal allergen sensitization and challenge to mice fed PBS on the same time course. Respiratory parameters were assessed by whole body unrestrained plethysmography and mechanical ventilation with forced oscillation. Bronchoalveolar lavage fluid (BAL) and lung homogenate (LH) were assessed for cytokines and chemokines by ELISA. BAL inflammatory cells were also collected and examined by light microscopy.

**Results:**

CRA feeding prior to allergen sensitization and challenge led to a significant improvement in respiratory health. Airways hyperreactivity measured indirectly via enhanced pause (Penh) was meaningfully reduced in the CRA-fed mice compared to the PBS fed mice (2.3 ± 0.4 vs 3.9 ± 0.6; p = 0.03). Directly measured airways resistance confirmed this trend when comparing the CRA-fed to the PBS-fed animals (2.97 ± 0.98 vs 4.95 ± 1.41). This effect was not due to reduced traditional inflammatory cell chemotactic factors, Th2 or other cytokines and chemokines. The mechanism of improved respiratory health in the tolerized mice was due to significantly reduced eosinophil numbers in the bronchoalveolar lavage fluid (43300 ± 11445 vs 158786 ± 38908; p = 0.007) and eosinophil specific peroxidase activity in the lung homogenate (0.59 ± 0.13 vs 1.19 ± 0.19; p = 0.017). The decreased eosinophilia was likely the result of increased IL-10 in the lung homogenate of the tolerized mice (6320 ± 354 ng/mL vs 5190 ± 404 ng/mL, p = 0.02).

**Conclusion:**

Our results show that oral tolerization to CRA can improve the respiratory health of experimental mice in a CRA-induced model of asthma-like pulmonary inflammation by reducing pulmonary eosinophilia.

## Background

Asthma is a significant chronic health problem in the U.S. and other developed nations. It accounts for millions of hospitalizations and thousands of deaths per year. The incidence of this burdensome ailment has increased by 50% every ten years raising the prevalence in the U.S. to 26.7 million in 1997 [1,2].

A significant disparity in new asthma diagnoses has been noted between urban and suburban/rural children. Urban children suffer a significantly higher incidence of asthma than their non city-dwelling counterparts [1]. This effect has been attributed to differences in exposure to sensitizing allergens present in the environment. Namely, it is theorized that an allergen(s) present at higher concentration, or in a unique combination in urban environments is the principal aggravating factor. Suspected allergens include feline, canine, murine, dust mite and cockroach allergen and pollutants such as diesel particulates.

A landmark paper published in 1997 identified a link between asthma incidence in urban children and antigens derived from the ubiquitous pest *Blattella germanica*, the German cockroach [3]. This link was further investigated in 1998 by the development of the first cockroach allergen induced model of asthma [4]. Publications from this lab have shown that aqueous house dust extract from the kitchens of severely asthmatic, urban children can induce allergic asthma-like symptoms in mice. Moreover, the most potent and abundant allergens identified in the dust extract were proteins of the German cockroach [5]. Thus, the study of cockroach allergen as an inducer of asthma in mice and humans is a rational first step in untangling the complex web of environmental exposure and allergic asthma.

Despite the alarming increase in prevalence and incidence of asthma in recent times, treatments have evolved slowly. Current standards of care include a long lasting anti-inflammatory agent such as an inhaled glucocorticoid or long acting β2 adrenergic agonist, combined with a short acting β2 agonist rescue inhaler and or epinephrine injections. Other effective agents include anti-IgE monoclonal antibodies, leukotriene receptor antagonists, mast cell degranulation inhibitors, antihistamines and cholinergic antagonists [1,6,7]. These treatments focus on alleviating the symptoms of the disease rather than addressing the cause. Specifically, they manipulate events occurring downstream of the aggravating stimulus to diminish certain aspects of the response. While these drugs are very effective at decreasing exacerbations and halting attacks, they tend to lose effectiveness over time. Asthma progresses because chronic, inflammation-induced lung remodeling coupled with drug desensitization, allow the disease to become more severe and recalcitrant to treatment over time.

Allergen desensitization is thus a highly attractive option for the treatment of allergic-type inflammatory diseases. This form of treatment has a significant advantage over standard therapeutic agents in that it addresses the fundamental cause of asthma rather than modifying downstream mediators. In addition allergen desensitization has the advantage of being tailored to the individual patient. A skin hypersensitivity panel can be performed for a wide array of potential allergens in order to identify the causative agent(s). Thus, the desensitization regimen can be narrowly focused on the most likely causative allergens, offering symptomatic relief based on rational and specific treatment targeted to the inciting allergens(s).

Current desensitization procedures deliver increasing titers of the putative allergen subcutaneously over a period of weeks. The patient must travel to their physician's office and remain in the clinic for 30 minutes after the injection in case a life-threatening reaction occurs. In addition, the administering health professional must be specially trained and have the capacity to treat severe anaphylaxis [8,9]. These factors make current desensitization procedures relatively costly and inconvenient for both the patient and the care provider.

Sublingual/oral allergen desensitization is starting to gain wider interest and acceptance[10,11] with significant advantages over subcutaneous desensitization. Chief among these is the lower potential for anaphylaxis. The patient is monitored for severe reactions only after the first dose; subsequently, he/she can self-administer treatment in their own home. This vastly increases compliance as there is very little disruption of the patient's daily schedule. This is especially true for asthmatic children in whom compliance is a significant issue. Therefore, oral desensitization is an ideal candidate for the treatment of allergic type asthma.

Although oral tolerance is gaining wider acceptance, the mechanism of how this occurs has not been determined. We designed studies to examine whether oral tolerance would effectively relieve asthma-like pulmonary inflammation in response to cockroach allergens, and determine the mechanism(s) of why oral tolerance is effective.

## Methods

### Experimental model

We used exclusively female HSD-ICR mice at 18-20 grams. (Harlan Sprague Dawley Inc., Frederick, MD). All data represent the combination of 3 replicates except for direct airways resistance measurement which is the combination of 2 replicates. All experiments were reviewed and approved by the Boston University School of Medicine Institutional Animal Care and Use Committee.

### Allergen feeding

Oral exposure to allergens was performed by gavage. Briefly, a solution containing 16 ug of combined Blag1 and Blag2 was prepared in 100 ul of PBS. Mice were lightly anesthetized with isoflurane and the solution delivered directly to the stomach by means of a metal gavage needle. Control mice received 100 ul of PBS by the same method. Feeding was performed daily for 4 days. The mice were given a 3 day rest period before the allergen sensitization protocol. For allergen specificity ovalbumin (OVA) was given on the same schedule and volume as those described above. The OVA solution was adjusted to provide the same total protein concentration as the CRA mixture, 7.35 mg/mL. Following the OVA tolerization period, the mice received CRA immunization and 2 CRA challenges along the same schedule as the CRA tolerized animals.

### Allergen sensitization

Cockroach antigen (CRA) was purchased from Greer Laboratories (Lenoir, NC) as a lyophilized whole body extract of the German cockroach *Blattella germanica*. The CRA was reconstituted in sterile PBS and the concentration of components Blag1 and Blag2 were assayed by ELISA. The concentration of the solution was adjusted so that 50 ul contained 8 ug of combined Blag1 and Blag2. The immunization (day 0) and 2 challenges (days 14 and 21) were delivered intratracheally by direct pharyngeal delivery which is subsequently inhaled [12]. Briefly, the mouse was suspended by its front incisors on an incline board, its tongue was gently pulled forward and the CRA solution was placed at the back of the pharynx in two 25 ul aliquots for aspiration. The immunization dose was a 1:2 of the stock solution and the challenges were a 1:4 containing 4 ug and 2 ug combined Blag1 and Blag2 respectively. The naïve mice received no CRA challenges. The 0 Hr mice were administered 2 intratracheal challenges of CRA and were assayed and sacrificed at the time they would have received their final allergen challenge, i.e. they did not receive the third challenge. The 1.5 Hr and 24 Hr mice were given the full set of 3 challenges and were assayed and sacrificed at 1.5 and 24 hours post final challenge respectively.

### Respiratory measurements

Mice were placed in unrestrained whole body plethysmograph chambers at the same time of day and exposed to a 2 minute aerosolization of PBS, 25 mg/mL or 50 mg/mL methacholine followed by a 5 minute recording period [13,14]. The mice were first allowed to explore the chambers with normal grooming behavior indicating that the mice had become acclimated. Direct resistance measurements were made on a flexivent instrument (ScireQ, Montreal, QC, Canada). Briefly, mice were anesthetized with pentobarbital, and the trachea was directly cannulated through a small incision. The mice were placed on the mechanical ventilator and then paralyzed with pancuronium bromide. A nebulizer attached to the instrument delivered PBS, and methacholine challenges at 25 and 50 mg/mL while airways resistance was measured.

### Sacrifice and Data Collection

The mice were anesthetized with intrperitoneal ketamine/xylazine and then sacrificed by exsanguination and cervical dislocation. The trachea was opened and cannulated with a length of flexible tubing and the lungs were lavaged with 2 mL of warm HBSS in 250 ul aliquots. The left lung was removed and fixed in 70% ethanol for histology. The right lung was placed into ice cold Complete Protease Inhibitor Cocktail (Roche Chemicals, Switzerland). The right lungs are then homogenized, the homogenate centrifuged at 10,000 G for 15 minutes and the supernatant removed for cytokine analysis. The cellular components of the whole lung homogenate were then resuspended in 0.5% cetyltrimethylammonium chloride (CTAC) and sonicated to release the contents of the eosinophilic granules. The supernatant of this mixture was collected and assessed for peroxidase activity (EPO). The lavage fluid was centrifuged at 600 G for 5 minutes and the supernatant was removed. The cell pellet was resuspended in 200 ul of RPMI, the red cells lysed and counted on Coulter particle counter (Beckman Coulter, Fullerton CA). The cells were then adhered to a slide and counted at 100 × magnification. The absolute cell counts per BAL sample were calculated for total white cells, neutrophils, macrophages, eosinophils and lymphocytes. Blood samples were taken from each mouse at exsanguination for cell counting on a Hemavet (Drew Scientific, Dallas, TX). Cell counts were expressed as the absolute number of a particular cell per 20 ul blood sample.

### Cytokine and chemokine analysis

Cytokines and chemokines were measured using sandwich ELISA [15]. Briefly, Nunc (Rochester, NY) plates were coated overnight at 4C with anti-cytokine antibodies, the plates were blocked, samples were incubated on plates for 2 hours at room temperature, a biotinylated secondary antibody was used to detect captured cytokines and chemokines following incubation with strepavidin conjugated horse radish peroxidase (SA-HRP) and a colorimetric reaction. BAL samples were diluted 1:2, lung homogenate samples were diluted 1:5 and standards contained an equal concentration of pooled naïve lung homogenate. Plates were read with a PowerWaveX plate reader (Bio-Tek Instruments, Hopkinton, MA).

### Statistics

Statistical comparisons were performed by two-tailed t-test in Graphpad Prism 4.0 (La Jolla, CA). Power analysis was performed using freeware tools on http://www.biomath.info/. The coefficient of variance was calculated as the ratio of the standard deviation and the mean of each data set or CV = standard deviation/mean.

## Results

### Respiratory Parameters

Initially, we examined the respiratory parameters in CRA-fed (tolerized) and PBS-fed mice following allergen sensitization and 2 challenges. We compared respiratory data in response to 50 mg/mL of methacholine (Mch) because this dose provided a maximal response. The first parameter we analyzed was enhanced pause (Penh). Penh is a dimensionless composite parameter which can be used to screen experimental animals for airways hyperreactivity to Mch and obstruction [16-18]. Changes in Penh can be caused by any factor which alters the caliber of conducting airways including inflammation, smooth muscle constriction and luminal obstruction with mucus.

**Figure 1:**
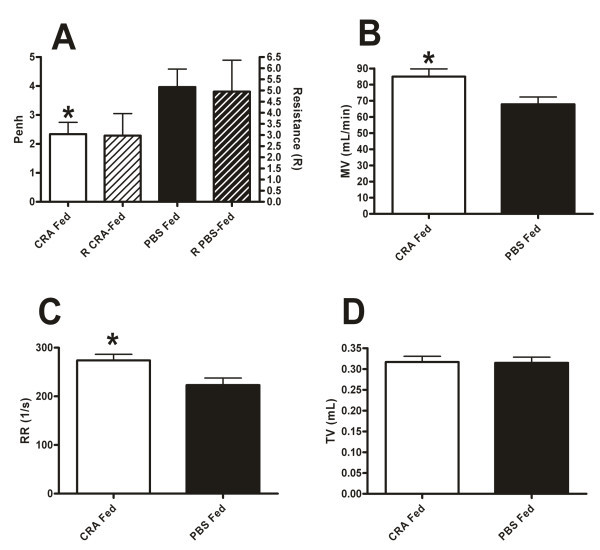
**Oral tolerance improves pulmonary respiratory parameters**. Penh and resistance (A) were measured in CRA fed and PBS fed mice 4 hours after final CRA challenge. Penh was recorded at the plateau of airways hyperresponsiveness, 50 mg/mL for 5 minutes for Penh and 25 mg/mL for 10 minutes for resistance. Minute ventilation (B), respiratory rate (C) and tidal volume (D) were measured in CRA fed and PBS fed mice 4 hours after final CRA challenge. Data were recorded for 5 minutes in response to 50 mg/mL of methacholine. Each value is the mean ± SEM for n = 18 (Figure 1A Penh, 1B, 1C, 1D) and n = 8 (Figure 1A Resistance). * = p < 0.05 comparing CRA fed to PBS fed mice.

The Penh values of the CRA Fed mice were found to be significantly lower than that of the PBS Fed animals (Figure 1A). Because the reporting of plethysmograph data in isolation is controversial, this result was further verified by employing a forced oscillation device (FO) to directly assess airways resistance [19-21]. In these FO experiments, we compared the airways resistance of the CRA or PBS fed mice in response to 25 mg/mL of methacholine because this represented the plateau of airways hyper-responsiveness. The CRA fed mice confirmed the trend towards decreased airways resistance which was not significant (Figure 1A). Power analysis revealed that 21 experimental animals per group would be needed to verify this difference in a statistically significant manner. This value is very close to the 19 animals per group which were needed to identify the difference using Penh. Finally, the directly measured resistance values in CRA and PBS fed mice were compared with the analogous Penh values using a ranked Pearson correlation. This analysis revealed a 93% correlation between resistance and Penh in the CRA fed mice and an 81% correlation in the PBS fed animals.

Next we examined a host of other respiratory parameters which we have found to be correlated with the respiratory health of mice in our model of asthma. First we examined minute ventilation (MV). The minute ventilation of the CRA fed mice was significantly higher than that of the PBS fed animals (Figure 1B). Maintenance of an elevated MV in times pulmonary distress is associated with superior respiratory health [22-24]. Namely, when lung disease is more severe, acute exacerbations cause involuntary decreases in MV. To determine the etiology of this difference we examined the components of MV, respiratory rate (RR) and tidal volume (TV). Respiratory rate was significantly higher in the CRA fed mice but there was no difference in TV (Figure 1C, D). This indicates that the low MV in the PBS fed mice was due principally to a depressed RR.

**Figure 2:**
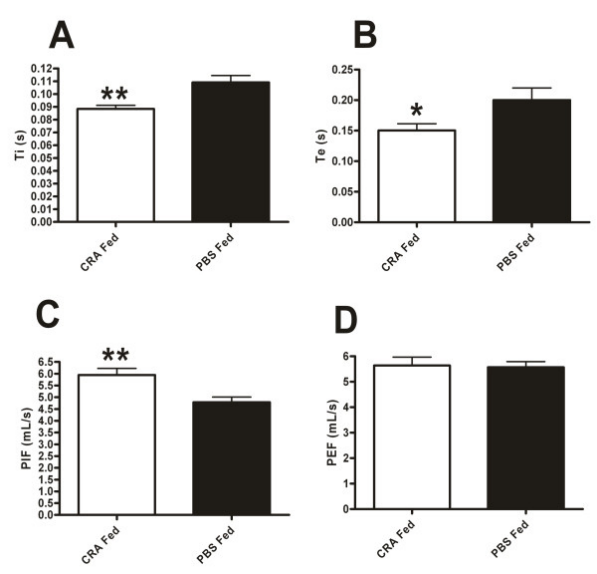
**Oral tolerance improves inspiratory and expiratory parameters**. Time of inspiration (A), time of expiration (B), peak inspiratory flow rate (C) and Peak expiratory flow rate (D) were measured in CRA fed and PBS fed mice 4 hours after final CRA challenge. Data were recorded for 5 minutes in response to 50 mg/mL of methacholine. Each value is the mean ± SEM for n = 18. * = p < 0.05 and ** = p < 0.01 comparing CRA fed to PBS fed mice.

Next we compared the time of inspiration (Ti) and expiration (Te) of the CRA or PBS fed mice. The orally tolerized mice displayed significantly lower Ti and Te when compared to the PBS fed animals (Figure 2A, B). Increased Ti and especially Te is associated with airways obstruction in inflammatory processes [25-28]. Finally we examined the peak inspiratory (PIF) and expiratory (PEF) flow rates which indicate the forcefulness of the inspiratory and expiratory cycles respectively. Serial measurements of PEF (peak flow) are often used to evaluate the effectiveness of asthma control regimens [25]. PIF was significantly depressed in the PBS fed animals as compared to the CRA fed mice while PEF showed no significant differences between the groups (Figure 2C, D). These data, considered together suggest that the CRA Fed mice are in a superior state of respiratory health and were better able to compensate upon exposure to methacholine (by increasing RR, and PIF and maintaining low Ti and Te) in order to maintain an elevated minute ventilation.

### Pulmonary Inflammation

**Figure 3:**
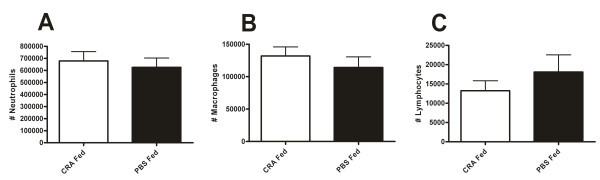
**Bronchoalveolar lavage (BAL) cellular constituents**. BAL neutrophils (A), macrophages (B) and lymphocytes (C) in CRA fed and PBS fed mice harvested 4 hours after final CRA challenge. There was no change in the number of these cells in the BAL. Cell counts were expressed as the absolute number of cells collected in each sample. Each value is the mean ± SEM for n = 18.

To determine whether this improvement in respiratory health was due to inhibition of inflammatory cell recruitment we analyzed the inflammatory cells present in the bronchoalveolar lavage fluid (BAL) of the experimental mice. First we examined the levels of neutrophils, macrophages and lymphocytes. Neutrophils are potent inflammatory mediators in asthma and typically arrive to sites of inflammation in a rapid fashion [29,30]. Macrophages are the only inflammatory cells typically present in the lungs and serve as immune surveillance [31]. Finally, lymphocytes are key components of the adaptive immune system and T-regs are thought to be important in the execution of immune tolerance to ingested antigen [32-34]. However, the counts of neutrophils, lymphocytes and monocytes were not significantly different in the lavage fluid of the CRA fed mice as compared to the PBS fed mice (Figure 3A-C).

**Figure 4:**
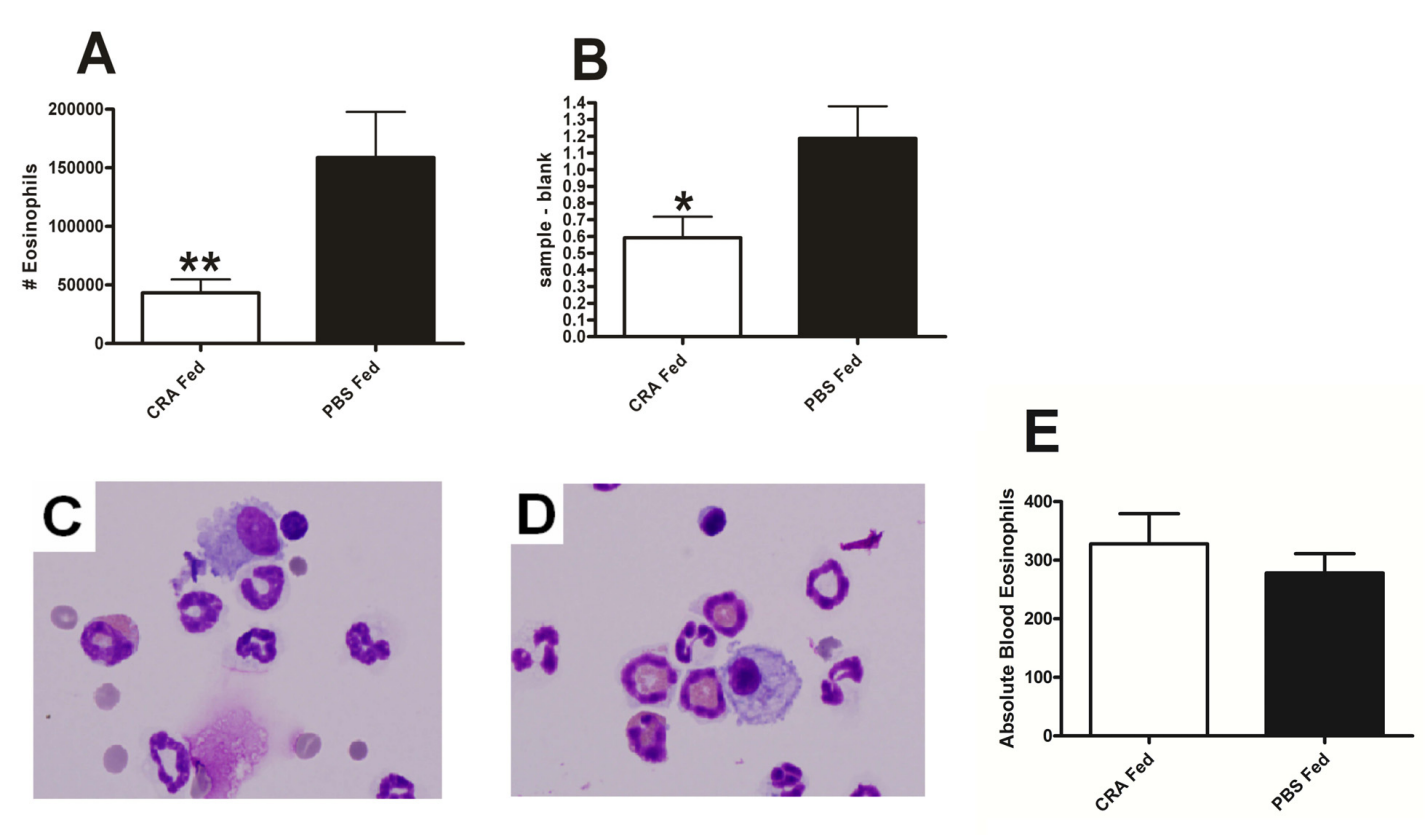
**Pulmonary and blood eosinophil recruitment**. Bronchoalveolar lavage eosinophils (A) and lung homogenate eosinophil specific peroxidase (EPO) activity (B) were measured in CRA fed and PBS fed mice 4 hours after final CRA challenge. EPO was assessed in the lung homogenate following lavage to collect cells. Representative cytospin images from CRA fed (C) and PBS fed (D) mice stained with H+E at 100×. The circulating blood levels of eosinophils (E) were assessed in CRA fed and PBS fed mice 4 hours after final challenge using a Hemavet. Each value is the mean ± SEM for n = 18. ** = p < 0.01 comparing CRA fed to PBS fed mice.

Lung eosinophilia is a hallmark of severe asthma. These cells respond to many chemotactic factors including the eotaxins (released from airways epithelial cells and macrophages among others) and the Th2 cytokines IL-4, 5 and 13 (released primarily from T cells) [35-37]. Eosinophil counts were significantly depressed in the CRA fed group (Figure 4A,C,D). We verified the diminished presence of eosinophils by measuring the eosinophil specific peroxidase (EPO) activity of the lung homogenate and found that it was significantly decreased in the CRA fed animals (Figure 4B). Finally, we measured the levels of circulating eosinophils in the blood using a Hemavet and found no statistically significant difference between the CRA-fed and PBS-fed groups of mice (Figure 4E) Blood eosinophil numbers are displayed as the absolute number of cells per 20 ul blood sample. Each value is the mean ± SEM for n = 18. ** = p < 0.01 comparing CRA fed to PBS fed mice.

**Figure 5:**
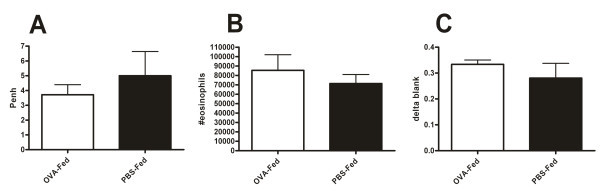
**Antigen specificity in oral tolerance**. Penh in response to 50 mg/mL of methacholine (A), bronchoalveolar lavage eosinophils (B) and EPO activity (C) in OVA and PBS fed, CRA immunized and challenged mice. Each value is the mean ± SEM for n = 8. None of these parameters were statistically different from each other.

We verified that the tolerization effect was antigen specific through OVA experiments. Mice were tolerized to OVA and then sensitized and challenged with CRA. There was no difference in the airways hyperreactivity as measured by Penh, bronchoalveolar lavage eosinophils or lung homogenate EPO levels (Figure 5A-C).

**Figure 6:**
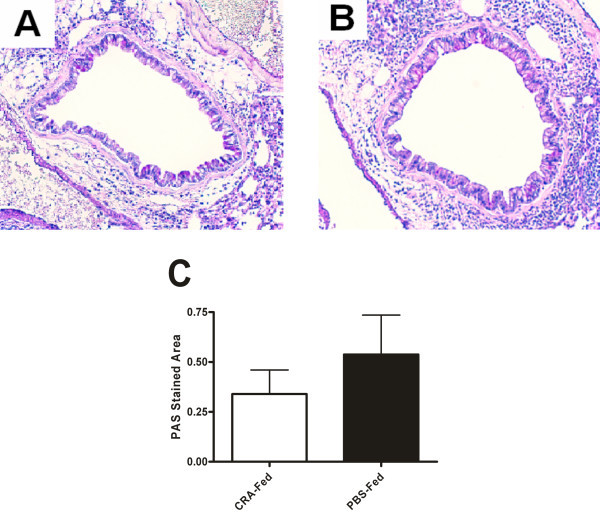
**Lung histology**. Representative lung histology sections from CRA-fed (A) and PBS-fed (B), CRA immunized and challenged mice. Sections are stained with H+E and PAS for mucus and magnified 10×. Quantitation of PAS staining area in CRA-fed and PBS-fed mice (C). Each value is the mean ± SEM for n = 18.

In order to determine a mechanism of depressed eosinophil recruitment in the CRA fed mice we examined a number of cytokines and chemokines in the bronchoalveolar lavage fluid (BAL) and lung homogenate supernatant (LH) (Table 1). The most obvious candidates, eotaxins 1 and 2 did not differ between the two experimental groups. Additionally, we found no significant differences in any of the Th2 cytokines (IL-4, IL-5 and IL-13) or in any of the mediators often associated with airways hyperreactivity such as TNF-α and IFN. To see if the depressed eosinophil recruitment could be related to antibody production, we measured serum IgG and IgE and found no significant difference between CRA and PBS fed mice. Additionally, histological analysis of whole lung sections with PAS stain revealed no difference in airways mucus levels between the experimental groups (Figure 6A-C).

**Table 1:** Parameters measured in CRA fed and PBS fed mice 4 hours after final challenge. BAL and LH cytokines assessed by sandwich ELISA and given in pg/mL.

	BAL			LH				Other		
	**CRA-Fed**	**PBS-Fed**	**p**	**CRA-Fed**	**PBS-Fed**	**p**		**CRA-Fed**	**PBS-Fed**	**p**

Eotax-1	77.86 ± 7.76	96.3 ± 7.41	0.09	7870 ± 2287	6496 ± 983.8	0.58	Tot.IgG	4.39 ± 0.77	3.25 ± 0.42	0.21

Eotax-2	173.3 ± 40.6	214.3 ± 47.9	0.54	8500 ± 1243	8340 ± 1651	0.94	Tot. IgE	45.21 ± 5.47	54.05 ± 8.92	0.41

IFN-γ	45.67 ± 8.3	59.95 ± 14.8	0.41	2951 ± 297.8	2981 ± 275.8	0.94	Mucus	0.34 ± 0.12	0.54 ± 0.19	0.52

RANTES	34.34 ± 3.48	41.96 ± 3.38	0.12	1461 ± 216.7	1341 ± 198.8	0.69	BAL N	6.8E5 ± 7.7E4	6.3E5 ± 7.7E4	0.63

MIP-2	69.06 ± 4.58	72.27 ± 6.13	0.42	4739 ± 650.7	4706 ± 656.8	0.97	BAL M	1.3E5 ± 1.4E4	1.1E5 ± 1.6E4	0.41

KC	816.7 ± 142.9	813.6 ± 89.40	0.98	11814 ± 2262	10698 ± 1750	0.70	BAL L	1.3E4 ± 2.5E3	1.8E4 ± 4.5E3	0.35

IL-4	86.20 ± 8.04	91.96 ± 9.90	0.65	3740 ± 612.6	3778 ± 629.7	0.97	MPO	1.43 ± 0.12	1.48 ± 0.11	0.74

IL-5	17.19 ± 2.18	17.18 ± 2.18	0.99	1992 ± 109.9	2042 ± 143.4	0.79				

IL-13	76.64 ± 6.83	91.34 ± 9.04	0.20	3859 ± 510.9	3916 ± 492.0	0.94				

IL-17	80.83 ± 13.56	103.4 ± 16.96	0.30	1991 ± 306.9	2094 ± 318.0	0.82				

TNF	431.1 ± 51.99	532.3 ± 67.78	0.24	1483 ± 115.3	1584 ± 81.05	0.48				

IL-12	116.8 ± 30.29	101.8 ± 25.72	0.71	4639 ± 795.6	4654 ± 825.2	0.99				

Total IgG and IgE given in ng/mL. Mucus reported as area of PAS staining following color deconvolution and quantitation by ImageJ freeware. BAL cells represented as absolute cells per 2 mL sample. MPO reported as sample absorbance minus blank absorbance. All values are the mean ± SEM for n = 18

**Figure 7:**
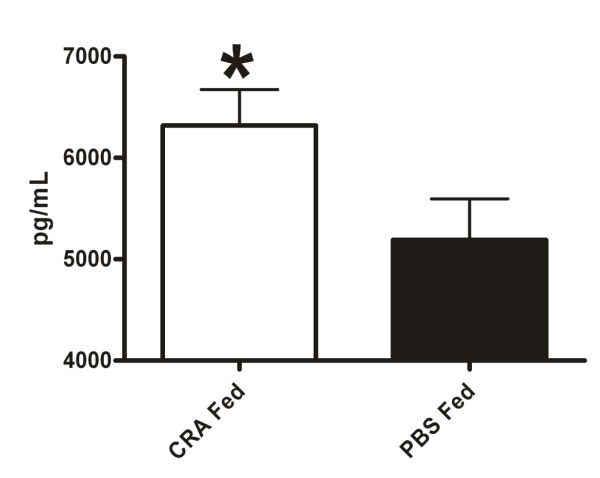
**Lung homogenate IL-10**. Oral tolerance increases pulmonary levels of IL-10. IL-10 was measured in the lung homogenate supernatant in CRA fed and PBS fed mice 4 hours after final CRA challenge. Cytokine concentration was assessed by sandwich ELISA with control lung homogenate supernatant background removed. Each value is the mean ± SEM for n = 18. * = p < 0.05 comparing CRA fed to PBS fed mice.

Finally, we examined IL-10 levels in the BAL and LH as this cytokine is known to inhibit eosinophil recruitment to the lung [38,39]. The BAL showed equivalent levels of IL-10 between the CRA and PBS fed mice. However, the lung homogenate supernatant of the CRA fed mice contained significantly elevated levels of IL-10 compared to the PBS fed animals (Figure 7). Additionally, the serum levels of IL-10 were below detection limit in both groups of mice (data not shown).

## Discussion

A number of research groups have reported amelioration of experimental allergic and autoimmune diseases through the establishment of oral tolerance [40-42]. Most of these studies employed well defined solitary antigens such as OVA in order to isolate the desired response without excessive background interference. Our current work differed from previous approaches in that it employed a complex allergen mixture containing the defatted whole body extract of German cockroaches. This mixture contains the complete corporeal proteins of the cockroach, as well as a host of bioactive enzymes and the innate immune stimulants chitin and LPS. These experiments represent a broader approach to allergen desensitization. Namely, this study sought to tolerize experimental animals to the whole host of cockroach derived products which human subjects are likely to encounter in urban environments.

It has previously been reported that oral exposure to OVA in the drinking water of experimental mice significantly decreased the airways hyperreactivity to methacholine and the production of Th2 cytokines such as IL-5 and IL-13 [43]. Our research obtained similar results to this previous study since the Penh of experimental mice fed with CRA was significantly lower than that of PBS fed mice. This indicated that the allergen fed mice were in a superior state of respiratory health as a result of the prior, gastric exposure to the putative antigen(s).

As mentioned previously, other research groups, using isolated allergens have been able to induce significant declines in Th2 cytokines following antigen tolerization. Interestingly, we saw no differences in any of the cytokines and chemokines traditionally measured in allergic pulmonary inflammation. We therefore had to seek other explanations for the improved respiratory health we observed in our allergen tolerized mice. Other groups have shown decreases in inflammatory cell infiltrate following oral allergen desensitization [44,45]. In our studies we observed significantly diminished eosinophil recruitment to the lung airspaces with no differences in the BAL levels of other inflammatory cells. However, circulating blood eosinophils did not differ between the experimental groups. That an equivalent number of eosinophils were recruited into the blood in the CRA-fed and PBS-fed animals is not surprising considering that BAL and LH levels of chemotactic agents for these cells were equivalent between groups. This suggests that the mechanism for decreased eosinophil infiltration into the lung air spaces is regulated at the level of organ itself, potentially during adhesion/transmigration. In addition EPO activity was also significantly decreased in the CRA fed mice. Taken together, these data suggest that the recruitment of eosinophils has been inhibited to both the bronchoalveolar space and the lung parenchyma as a whole.

Although the numbers of eosinophils in the lung were decreased in the CRA fed mice, the levels of chemotactic agents were not significantly different between the two groups. Eotaxin 1 and 2 are as their name suggests potent chemo attractants for eosinophils yet their concentrations were not significantly decreased in the CRA fed groups [46,47]. IL-4 and IL-5 are also known eosinophil chemotaxins and activators, but the levels of these cytokines were equivalent between the experimental groups [48,49]. In addition, IL-13 which has been identified as necessary for the entrance of eosinophils into the lung did not differ between the groups [50,51].

Having exhausted the traditional mediators of eosinophil recruitment we decided to probe IL-10 levels. This cytokine has been shown to inhibit the recruitment of eosinophils and alternately improve or aggravate airways hyperreactivity [38,39,52]. IL-10 levels were significantly elevated in the lung homogenate supernatant of the CRA fed mice with the means differing by approximately 1000 pg/mL. This represents a 20% increase in IL-10 production in the allergen fed mice. Whether this is a biologically significant difference is uncertain. IL-10 is known promote the development of oral tolerance, but the elevated levels seen after the final challenge are not directly related to the tolerization period which took place 24 days prior to the final challenge [53,54]. This supposition is supported in that the OVA tolerized mice did not differ in lung homogenate IL-10 levels as would be expected if a gastric tolerizing event was responsible for this late cytokine production (data not shown). Thus it seems likely that the post challenge increase in IL-10 is a separate event related to the pulmonary allergen exposure. Interestingly, the secretion of this immunomodulatory cytokine seems limited to the pulmonary environment as serum levels of IL-10 were below detection limit.

In conclusion, oral exposure to cockroach allergen prior to pulmonary sensitization and challenge leads to significantly improved respiratory health in experimental mice. This improvement is due to reduced eosinophil recruitment into the air spaces and lung parenchyma. The inhibition of eosinophil recruitment may be related to increased production of IL-10 in the lung. Finally, this research suggests that oral tolerization to a complex environmental allergen is a viable option for desensitization in allergic airways disease.

## Conclusion

This research indicates that oral tolerization is a valid means to reduce pulmonary inflammation in a mouse model of allergic asthma. Oral tolerization presents an attractive therapeutic option for human asthmatics in that it addresses the primary cause of allergic asthma exacerbations rather than simply blunting the symptoms. In addition oral tolerization offers significant advantages over other desensitization procedures (epidermal injections) in that there is a much lower risk of anaphylaxis and treatments may be self-administered after an initial observation period.

## Competing interests

The authors declare that they have no competing interests.

## Authors' contributions

Vaickus, L.J. performed most of the data collection and analysis.

Bouchard, J. provided data and made contributions to study design.

Kim, J. provided data and made contributions to study design.

Natarajan, S. provided data and made contributions to study design.

Remick, D.G. is the principal investigator and mentor of the 1^st ^author and made contributions to study design.

All authors have read and approved the final manuscript.
